# Regulatory Snapshots: Integrative Mining of Regulatory Modules from Expression Time Series and Regulatory Networks

**DOI:** 10.1371/journal.pone.0035977

**Published:** 2012-05-01

**Authors:** Joana P. Gonçalves, Ricardo S. Aires, Alexandre P. Francisco, Sara C. Madeira

**Affiliations:** 1 Knowledge Discovery and Bioinformatics Group (KDBIO), INESC-ID, Lisbon, Portugal; 2 Instituto Superior Técnico, Technical University of Lisbon, Lisbon, Portugal; Semmelweis University, Hungary

## Abstract

Explaining regulatory mechanisms is crucial to understand complex cellular responses leading to system perturbations. Some strategies reverse engineer regulatory interactions from experimental data, while others identify functional regulatory units (modules) under the assumption that biological systems yield a modular organization. Most modular studies focus on network structure and static properties, ignoring that gene regulation is largely driven by stimulus-response behavior. Expression time series are key to gain insight into dynamics, but have been insufficiently explored by current methods, which often (1) apply generic algorithms unsuited for expression analysis over time, due to inability to maintain the chronology of events or incorporate time dependency; (2) ignore local patterns, abundant in most interesting cases of transcriptional activity; (3) neglect physical binding or lack automatic association of regulators, focusing mainly on expression patterns; or (4) limit the discovery to a predefined number of modules. We propose Regulatory Snapshots, an integrative mining approach to identify regulatory modules over time by combining transcriptional control with response, while overcoming the above challenges. Temporal biclustering is first used to reveal transcriptional modules composed of genes showing coherent expression profiles over time. Personalized ranking is then applied to prioritize prominent regulators targeting the modules at each time point using a network of documented regulatory associations and the expression data. Custom graphics are finally depicted to expose the regulatory activity in a module at consecutive time points (snapshots). Regulatory Snapshots successfully unraveled modules underlying yeast response to heat shock and human epithelial-to-mesenchymal transition, based on regulations documented in the YEASTRACT and JASPAR databases, respectively, and available expression data. Regulatory players involved in functionally enriched processes related to these biological events were identified. Ranking scores further suggested ability to discern the primary role of a gene (target or regulator). Prototype is available at: http://kdbio.inesc-id.pt/software/regulatorysnapshots.

## Introduction

Gene regulation is the major orchestrator of cellular activity, directing the creation of proteins designed to participate in every biological process. Considerable effort has been undertaken to unveil regulatory mechanisms and advance the knowledge on complex system responses and dysregulation events leading to medical conditions. In particular, transcription has been extensively studied for its essential role in gene regulation, determining which genes should be transcribed into mRNA and influencing their expression rates. Explaining the translation of a biochemical stimulus into a cellular outcome is however a challenging task. One of the reasons is that most transcriptional responses result from a concerted action of multiple transcription factors (TFs). Regulatory players are often involved in diverse pathways simultaneously or over time. Additionally, mechanisms such as dynamic feedback loops, add layers of complexity as they generate intricate responses with transient gene products frequently found in regulatory cascades.

Individual regulatory associations have been actively predicted using diverse techniques, from ChIP-chip experiments to automated assessment of TF-binding site affinity. Transcriptional responses have also been investigated looking for significant changes and patterns. Nonetheless, research has been progressively evolving toward the study of organisms from a systemic standpoint and the next endeavour is to assemble heterogeneous elementary data into functional representations of regulatory networks, considering both control and behavior, to support the modeling and prediction of system’s responses to specific conditions. Available computational solutions usually fit into one of two groups. Reverse-engineering, also termed network inference, regards the system as a mathematical function with parameters. Models such as bayesian networks or differential equations are fitted to the experimental data using learning algorithms [1]–[3]. Alternatively, a mining perspective motivates the identification of functional components, or modules, considered as the basic building blocks of regulatory networks [4], [5]. This modular organization of biological systems has been defended under the assumption that their design resembles the architecture of complex computational and communication systems [5]–[7]. Hybrid methods combine mining and inference in an integrated optimization approach where each technique feeds the other complementary information [8], [9].

Several authors have addressed the module identification problem [4], [10]–[17], focusing mainly on structural or static properties [4], [10]–[12]. Nevertheless, regulatory activity is described by a series of events pertaining a particular order, relevant to the outcome. Notably, the ability to monitor transcriptional trends and to observe the emergence of patterns in expression time series can provide important insights into regulation dynamics [18]. A number of module identification methods proposed to date considers expression time series [13]–[17], [19]–[21]. Yeang and Jaakkola [13] compute latencies in transcription activation using a TF-target graph and expression time series and then apply greedy clustering to group genes bound by common TFs. Similar methods perform time-delay analyses to reveal associations between expression profiles of TFs and targets [22], [23], not necessarily addressing the identification of modules. In these cases, physical binding is not enforced and inferring regulations by aligning expression profiles may lead to a large number of false positives, as coherent profiles are a characteristic of co-regulation rather than TF-target association. Kasabov et al. [14] model gene regulatory networks as discrete-time approximations of differential equations and expression trajectories using a Kalman filter, and apply a genetic algorithm to group genes in modules. Discovery is limited to modules of predefined size, which may present a major drawback when prior knowledge is unavailable. Wu et al. [16] integrate expression, ChIP-chip, binding sites and mutant data. Stress-responsive targets, regulations and TFs are identified according to numerical criteria and statistical tests based on expression fold change, evidence of physical binding, and portion of stress-responsive targets of TF sets. Transcriptional coherence in a module is not ensured, as expression is only used to predict regulations. Zhang et al. [19] reverse engineer the modules through fuzzy c-means clustering of expression and functional category data. Particle swarm optimization and recurrent neural network are used to derive relations between modules, although disregarding the chronological order of events. Alternative methods identify modules based on expression profile correlation [17], [20], [21], potentially combined with functional enrichment [17], [20], but ignore physical binding. Three recent tools, developed by Segal et al. [8], Novershtern et al. [9], and Kundaje et al. [1] focus on reconstructing regulatory pathways underlying measured responses, using physical interactions and expression. They fit either Bayesian models [8], [9] or alternating decision trees [1] to the data under the assumption that the expression of the targets correlates or can be predicted from that of their regulators, which is known not to be true in most cases. Additionally, these techniques rely heavily on prior knowledge. Exploring well characterized and isolated data relating to the biological process under study is likely to provide a competitive advantage in performance over other approaches, besides circumventing the typical computational intensiveness and poor scalability of these methods. Nevertheless, it will also prevent the analysis or hamper de novo discovery in not so well studied biological processes.

Most of the strategies revised herein rely on clustering techniques to unravel transcriptional trends, searching for global patterns. It has been often recognized that clusters are not able to describe the complex nature of transcriptional response, as genes tend to behave coherently only in specific time frames and may be involved in different functional groups over time [1], [24]. Local patterns are particularly relevant when analyzing expression over time, given that biological processes are expected to occur within time frames. Notably, biclustering effectively addresses the discovery of these signals and efficient techniques have been proposed for the special case of expression data with a temporal dimension. [17], [18], [24].

We propose Regulatory Snapshots, a computational framework to identify regulatory modules from expression time series and regulatory associations. First, we unravel sets of genes exhibiting coherent expression profiles using a state of the art temporal biclustering method, CCC-Biclustering [17]. CCC-Biclustering takes advantage of reasonable biological assumptions in time series to convert the otherwise NP-hard biclustering formulation into a tractable problem [24]. It finds maximal transcriptional patterns spanning time intervals using string processing techniques based on suffix trees. Second, we identify relevant TFs targeting the genes in the transcriptional module at each time point. The personalized ranking method TFRank [25], originally proposed to prioritize TFs for a group of genes of interest, is applied to time series as follows. We diffuse an initial signal corresponding to the expression levels of the genes in the module at each time point through the transpose of the regulatory network graph and thus compute relevance scores for each TF, generating one ranking per time point. Third, we introduce the concept of regulatory snapshot to visually highlight the variation of relevant regulations over time in a module by exposing both the topology discovered via biclustering and the TF importance unveiled through prioritization.

Advantages of this framework include ability to: (1) combine mechanics as documented evidence of regulation defining the network topology (prior knowledge), and dynamics as transcriptional responses yielded by expression (experimental data); (2) search for local patterns, known to prevail in transcriptional response; (3) capture coordinated activity through algorithms specifically incorporating the temporal dimension; (4) identify relevant TFs relying on whole-network analysis, where transcriptional behavior is seen as a result of intricate system connectivity, rather than the direct action of a few players; (5) visually expose the variation of regulatory interactions relevance over time.

We assess the effectiveness of Regulatory Snapshots to identify regulatory modules underlying *Saccharomyces cerevisiae*’s response to heat shock [26] and human epithelial-to-mesenchymal transition [27]. In particular, we investigate the ability of our method to report biologically sound modules, characterized by (1) coherent transcriptional activity and functional relatedness of its genes, together with (2) regulations by TFs known to be involved in the biological processes enriched for the modules and undergone by the cells.

## Methods

**Figure 1:**
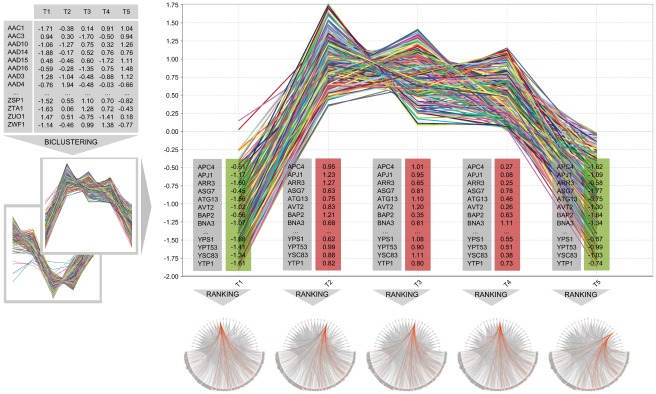
Regulatory Snapshots method. This figure shows an overview of the proposed method, Regulatory Snapshots, to mine regulatory modules from expression time series and regulatory associations in two steps. First, biclustering is applied to expression time series to find transcriptionally coherent genes and group them in transcriptional modules (biclusters). A personalized ranking strategy is then used to compute relevance scores for the transcription factors targeting the genes in the biclusters at each time point. Finally, regulators are sorted by relevance and a graphical representation, termed regulatory snapshot, is depicted to expose the architecture of the regulatory module.

In this section we describe Regulatory Snapshots, an integrative mining approach to identify regulatory modules over time. We define a regulatory module as a group of genes exhibiting coherent transcriptional activity in a given time frame and sharing a common set of regulators. In this context, we propose to discover and characterize regulatory modules involved in specific transcriptional responses in two steps (Figure 1). First, a time-aware biclustering algorithm is applied to the expression matrix to unravel groups of genes showing coherent temporal expression patterns (transcriptional modules). Second, a personalized ranking strategy is used to identify and prioritize the TFs targeting the transcriptional modules at each time point. Third, transcriptional modules and regulators are combined to form regulatory modules. Visual representations, termed regulatory snapshots after the method’s name, are finally depicted to expose the variation of relevant regulations in a module along the consecutive time points. Prototype software is currently available at: http://kdbio.inesc-id.pt/software/regulatorysnapshots.

### Temporal Transcriptional Module Identification

**Figure 2:**
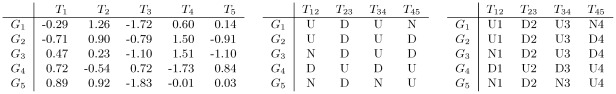
Transcriptional module identification: discretization and alphabet transformation. Illustrative example of discretization and alphabet transformation for a time series gene expression matrix: (left) original expression matrix *M*'; (center) discretized matrix *M*, obtained by applying a discretization based on transitions between time points to the original matrix *M*' using a three-symbol alphabet 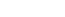 [28]; and (right) matrix *M* after the alphabet transformation that appends the column number to every symbol in the matrix.

Coherent transcriptional responses are sought using a biclustering algorithm, whose aim is to identify a set of biclusters satisfying particular characteristics of homogeneity [24]. For time series gene expression data, we adopt the concept of contiguous column coherent bicluster (CCC-Bicluster) and apply CCC-Biclustering to discover groups of genes with coherent expression profiles [17]. This algorithm uses a discretized version of the original expression matrix as follows. Let 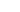 be an expression matrix defined by a set of genes (rows), 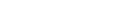, and a set of time points (columns), 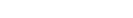, where 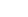 represents the expression of gene 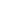 in time point 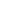. Expression levels in *M*' are discretized to a set of symbols, Σ, representing distinct activation levels in a new matrix *M*. Any discretization is eligible. In this work, we followed the original approach [17], [28] to convert matrix *M*' into *M*, where 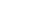 reflects the transition trend between the expression states of gene 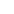 in time points 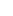 and 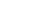, respectively. Alphabet 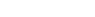 is used in this context, where *D*, *N*, and *U* mean *down-trend*, *no-trend* and *up-trend* (Figure 2).

**Figure 3:**
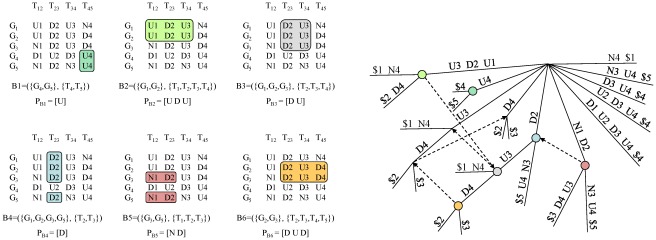
Transcriptional module identification: maximal biclusters. This figure shows all transcriptional modules, or maximal CCC-Biclusters with at least two rows, obtained by applying CCC-Biclustering to the transformed matrix in Figure 2. Maximal CCC-Biclusters are represented: (left) in the transformed discretized matrix of Figure 2; and (right) in the generalized suffix tree built for the strings in the rows of this matrix.

A CCC-Bicluster, 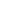, is defined as a subset of genes 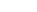 and a subset of contiguous time points 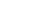 such that 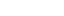, 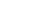 and 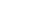, that is, every gene in *I* shares the same expression pattern spanning the consecutive time points in *J*. A CCC-Bicluster is maximal (Figure 3) if adding rows to *I* violates the coherence of the expression pattern (row-maximality) and adding a symbol to the beginning or end of the expression pattern induces changes in *I* (left-/right-maximality). CCC-Biclusters pertaining a single row are biologically uninteresting and are thus discarded.

In order to find all maximal CCC-Biclusters, CCC-Biclustering first performs a simple alphabet transformation to append the column number to each symbol in the discretized matrix (Figure 2). Regarding the rows of the transformed matrix as strings, a generalized suffix tree 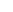 [29] is then built in order to match the common patterns and identify the maximal CCC-Biclusters. Such identification relies on the following relation between maximal CCC-Biclusters and nodes in 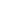: every right and row-maximal CCC-Bicluster with at least two rows corresponds to one internal node in 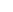 and every internal node in 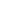 corresponds to one right and row-maximal CCC-Bicluster with at least two rows. Right- and row-maximality of the bicluster identified by an internal node *v* are guaranteed by generalized suffix tree construction. Left-maximality of an internal node *v* is guaranteed when either *v* has no incoming suffix links [29] or it has incoming suffix links only from nodes for which the number of leaves in their subtree is equal to the number of leaves in the subtree rooted at *v*. CCC-Biclustering uses efficient techniques to find these nodes in 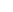 and report all maximal CCC-Biclusters in time linear on the size of the expression matrix. Figure 3 shows the relation between nodes in 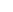 and maximal CCC-Biclusters using the illustrative example in Figure 2.

### Transcription Factor Prioritization

Relevant TFs are identified and prioritized through the application of personalized ranking to a network of regulatory associations. Such network can be described as a directed graph 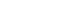. The set of vertices, *V*, is composed of regulators and target genes, while the set of edges, *E*, includes the regulatory associations between elements in *V*. Let *A* and *D* denote the adjacency and diagonal matrices of *N*, respectively, where 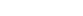 is the weight of edge 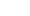 between regulator *u* and target gene *v* and 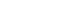 is the sum of weights of the outgoing edges of *u*. Given a set of initial target genes, or seeds, 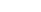, corresponding to the genes in a particular bicluster, we aim at obtaining a ranking on 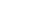, where *R* is the set of transcription factors regulating the set *S* of target genes. Personalized PageRank [30] is the most widely known approach to address the related problem of expressing web page quality surrounding particular pages of interest rather than over the entire Web, also known as local clustering on graphs [31], [32]. It involves a preference vector indicating the relevance of pages of interest, which can be regarded as the probability distribution of the seeds. The preference is then diffused through the web graph using a random walk based on a jumping constant denoting the probability of returning to the source nodes, called back probability.

We have previously relied on a related technique based on the heat kernel rank [32] to prioritize transcription factors exerting control upon a group of genes of interest, TFRank [25]. In this work, we apply TFRank to the case of time series aiming to identify the most relevant transcription factors targeting the transcriptionally coherent genes in a bicluster (transcriptional module) over time. This is achieved by initializing the preference vector with the expression levels of the genes in the bicluster at each particular time point and diffuse them using a random walk based on the heat kernel rank, recently shown to perform better in comparison to PageRank [32]. Formally, given a network graph *N*, the transition probability matrix *W* of a typical random walk on *N* is defined as 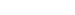. We further define 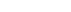, different from the Laplacian 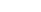 and its normalized form 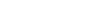. Note that *N* is a directed graph and *L* is normalized against the sum of weights of the outgoing edges. Given a preference vector *p*_0_ and a non-negative heat diffusion coefficient *t* to control the rate of diffusion and preference for closer or farther regulations, the ranking vector *p*_t_ is given by
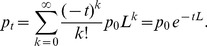
(1)


The discrete heat kernel [33] is a symmetric version of 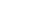. We use the discrete approximation [34]

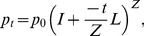
(2)


where *Z* is the number of iterations. The preference vector *p*_0_ contains the expression values of the target genes in *S*, as follows:
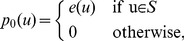
(3)


where *e*(*u*) is the expression value of gene *u*. To be able to reach the regulators from the targets, the signal is propagated through the regulatory network by traversing the regulations in reverse direction, or using the transpose of the network graph *N*. The procedure is repeatedly applied to generate one ranking per time point.

### Regulatory Module and Snapshot Representation

Coherent temporal transcriptional responses can be studied using current software tools [35]–[37], which enable the preprocessing of expression data, cluster or bicluster identification and post-processing of results, including functional enrichment analysis. Available applications for expression time series rely not only on knowledge discovery methods, but also on informative graphics of data and results. Similarly, we aim at picturing a regulatory module and its variation over time to support our exploratory analysis. We thus introduce the concept of regulatory snapshot to expose the architecture of a regulatory network while capturing the most relevant regulations targeting the genes in a regulatory module at a given time point. In this work, the functionality of the graphical tool devised by Aires et al. [38] has been extended to picture regulatory snapshots for temporal biclusters or transcriptional modules. Visually, a regulatory snapshot is a double semi-circled graph composed by top and bottom halfs of different radii, where the former displays TFs from left to right in decreasing order of a given score, and the latter contains the corresponding target genes. Regulations appear in the form of arcs in-between the outside semi-circumferences. User interaction enables to highlight the regulations for a particular node or set of nodes, which are then displayed in different color according to whether they encode for a “regulates" or “regulated by" association. In this case, we use the relevance score obtained for each TF by diffusing the expression values of the genes in a regulatory module at a given time point using personalized ranking. Several figures are captured to enhance the variation of the relevance of a given TF or set of TFs over time, thus the notion of snapshot. Additionally, we control the complexity of each figure by imposing an appropriate threshold on the scores of the regulators.

## Results and Discussion

In this section, we investigate the regulatory modules obtained using Regulatory Snapshots in two case studies. The first study concerns *Saccharomyces cerevisiae*’s response to heat shock upon exposure to 37°C. It focuses primarily on the biological soundness of the top transcription factors and their relevance over time, output by Regulatory Snapshots, for biclusters whose value has been previously confirmed [17]. The second study addresses the de novo discovery of regulatory modules underlying human epithelial-to-mesenchymal transition, where both discovered transcriptional response and control are investigated for functional coherence and enrichment. Finally, we compare our method with a state of the art algorithm for regulatory module discovery, Physical Module Networks [9].

### Yeast Response to Heat Stress

We analyzed time series expression data from yeast cells upon exposure to heat shock, measured at five time points (0’, 5’, 15’, 30’, and 60’) over a one hour period [26]. The following preprocessing was applied [17]: removal of genes with missing values or absent from the Saccharomyces Genome Database (SGD) [39]; normalization of expression values by gene to zero mean and unit standard deviation; and discretization expressing transitions between time points [28]. Regulatory associations from the YEASTRACT database [40] were used to build a graph comprising 6911 genes and 42690 interactions. In the following subsection we describe the application of Regulatory Snapshots to these data. We also investigate: (1) the positions achieved by TFs known to participate in the regulation of each module; (2) the biological relevance of top ranked TFs; (3) the variation of TF relevance over time and potential influence in the behavior of the targets; and (4) correlation between expression of TFs and targets.

#### Yeast heat stress regulatory snapshots

**Figure 4:**
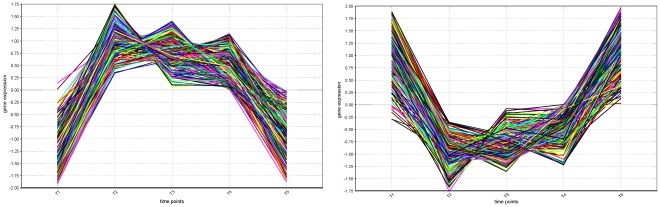
Expression profiles of yeast heat shock regulatory modules. This figure shows the expression profiles of the genes targeted in the regulatory modules 39 (left) and 151 (right) obtained for the yeast heat shock expression data and the yeast regulatory network containing regulations from the YEASTRACT database. Expression levels were normalized by gene to zero mean and unit standard deviation.

CCC-Biclustering was applied to the expression data, reporting 167 CCC-Biclusters with coherent responses (see Methods). For each bicluster, a pattern *p*-value was computed under the null hypothesis that a similar pattern would occur by chance in an expression matrix of equal size [17]. We filtered biclusters with Bonferroni corrected pattern *p*-value above 1 percent level and biclusters overlapping with a Jaccard similarity larger than 25 percent. Functional enrichment was assessed through a *p*-value based on the hypergeometric distribution. We considered highly significant all GO terms with a Bonferroni corrected *p*-value lower than 0.01. Six of the resulting biclusters, describing transcriptional upregulation (biclusters 39, 27 and 14) and downregulation (biclusters 147, 151 and 124) patterns, have been previously subject to biological analysis [17]. We focused on biclusters 39 and 151 as representatives of their categories (Figure 4), based on four key criteria: (1) coherence of transcriptional behavior spanning the largest time intervals, thus interesting from a temporal analysis standpoint; (2) significance of expression profiles, assessed by the bicluster pattern *p*-value; (3) presence of abrupt variations; and (4) interestingness of expression pattern, including evidence of anti-correlation between the two biclusters.

TFRank was applied to propagate the normalized expression levels of the genes in each bicluster through the transpose of the graph and identify the most relevant TFs at each measured time point (see Methods). We parameterized the method with 100 iterations and a heat diffusion coefficient value of 0.25 to moderately favor proximal regulators. Edges were not differentiated using weights based on the supporting evidence of each regulation, as this information is often biased toward well studied genes. Since the ranking score is additive, absolute values of expression were used. To evaluate the implications of not being able to discern positive from negative expression levels, we checked the genes in both sets. For every time point except the first, one of the sets would always include all genes. At 0’, only 2 and 9 genes were respectively positively and negatively regulated in biclusters 39 and 151. We decided to disregard their contribution to the ranking at this time point. Additionally, 15 and 11 genes were absent from the regulatory network and were excluded as well. Regulatory snapshots were generated to investigate relevant regulations at 0’, 5’, 15’, 30’ and 60’.

#### Yeast heat stress underlying regulation

**Figure 5:**
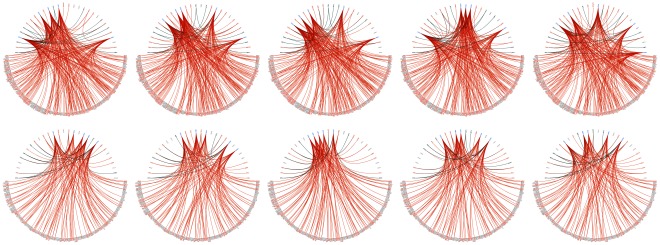
Regulatory snapshots of documented regulators in yeast heat shock modules. This figure shows regulatory snapshots obtained for yeast heat shock stress regulatory modules 39 and 151 over time (0’, 5’, 15’, 30’ and 60’), highlighting the ranks and interactions of regulators reportedly targeting the genes in these modules. Each snapshot along a row was obtained for a particular time point. Regulators and target genes are respectively represented in the top and bottom semi-circles, and regulators appear from left to right in decreasing order of ranking score. Orange and green arcs respectively identify “regulates" and “regulated by" relations for the highlighted regulators in each figure. The figures in the top row expose the ranks of Arr1p, Hsf1p, Msn2p, Rpn4p and Sok2p for bicluster 39, while the ones in the bottom row highlight the ranks of Arr1p, Ino4p, Mbp1p, Rpn4p and Swi4p for bicluster 151.

Bicluster 39 includes genes whose expression was abruptly upregulated during the first 5 minutes of exposure to heat, followed by residual variation between 5’ and 30’ and a large decrease in the last 30 minutes (Figure 4). Arr1p, Hsf1p, Msn2p, Rpn4p and Sok2p have been described to regulate the targets of this bicluster in an unspecific wide initial response to stress upon heat shock [17]. They promote an early activation of signaling cascades and other TFs involved in the transcriptional machinery mediating stress-specific reactions in subsequent time points. These five TFs appeared consistently among the top 30 for every time point (Figure 5). Msn2p was always in the top 15 and Sok2p repeatedly ranked above position 9, among 171 TFs. The regulatory snapshots for bicluster 39 show that, individually and together, Arr1p, Hsf1p, Msn2p, Rpn4p and Sok2p regulate a large percentage of genes in the bicluster and are regulated by most of the remaining TFs in the top 30 (Figure 5). This presents evidence of the intricate regulation promoted by signal transduction in transcriptional cascades. Most of these five TFs achieved their best score in the initial time points, supporting the reasoning that they could be involved in the abrupt expression increase. Bicluster 151 includes genes considerably downregulated during the first 5 minutes of exposure to 37°C. Their expression increased slightly from 5’ to 30’ and then abruptly in the last 30 minutes (Figure 4). Functional enrichment [17] has reported cell cycle repression in agreement with growth arrest upon sudden exposure to heat, involving the following TFs: Arr1p, Mbp1p, Ino4p, Rpn4p and Swi4p. Their relevance remained relatively stable over time (Figure 5). Nevertheless, all except Swi4p achieved highest ranks at 15’, when they further ranked tightly together. Interestingly, the relevance of Mbp1p and Swi4p exhibited coherent variation over time, supporting known cooperation in complexes with Swi6p to regulate cell cycle G1-S progression. Swi4p always ranked in the top 15, while Mbp1p and Ino4p consistently appeared within the most relevant 20. Arr1p and Rpn4p were in the first 25, from a total of 163 TFs targeting bicluster 151. These results confirm the ability of Regulatory Snapshots to automatically recover previously confirmed regulators, selected by manual/expert inspection.

Many players with potentially relevant roles were found in the top 20 (Figure 5). Sfp1p and Yap1p consistently ranked first and second for both biclusters. Sfp1p is known to control the expression of ribosome biogenesis genes in response to nutrients and stress [39] and has been specifically implicated with heat shock in the literature [41]. Yap1p is involved in tolerance to oxidative stress, which has been related to heat-induced cell death in yeast [42], [43]. For bicluster 39, several heat-responsive TFs arose. Hsf1p, a trimeric heat shock transcription factor binding DNA at variable heat shock elements and activating multiple genes in response to hyperthermia [39], ranked best at 0’ in position 14. Msn2p, binding DNA at stress response elements and inducing the expression of stress-responsive genes [39], ranked best at 15’ in position 9. Also Rpn4p achieved rank 11 at 15’. Sip4p, with specific RNA polymerase II transcription factor activity and regulation of transcription from RNA polymerase II promoter [39], ranked best at 30’ in position 7. Sip4p is also involved in the positive regulation of gluconeogenesis and invasive growth in response to glucose limitation [39], and has been implicated together with Mig1p in the activation of Hsf1p under glucose starvation conditions [44]. Crz1p, an activator of stress-responsive genes [39], ranked best at 15’ in position 17. For bicluster 151, interesting TFs were also retrieved. Hcm1p, a forkhead transcription factor driving S-phase specific expression of genes involved in chromosome segregation [39], ranked higher at 5’ in position 4. Yox1p, a homeodomain-containing transcriptional repressor [39], was considered most relevant at 30’ in position 13. Its ability to inhibit transcription agrees with the downregulation pattern described by the target genes. Also annotated with negative regulation of transcription from RNA polymerase II promoter [39], Abf1p and Kar4p achieved the best ranks 3 and 11 at time points 15’ and 0’, respectively. Similarly to Hcm1p and Yox1p, Kar4p is further involved in the regulation of mitotic cell cycle [39], in accordance with the highly significant GO terms reported for bicluster 151 [17].

**Figure 6:**
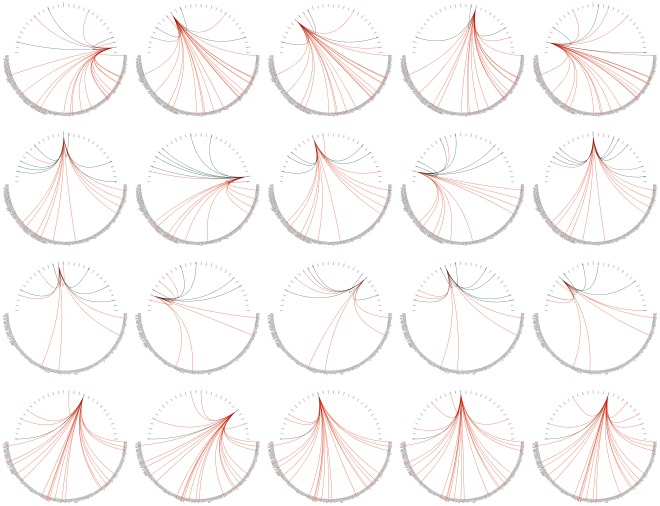
Regulatory snapshots of regulators with large relevance variations in yeast heat shock modules. This figure shows regulatory snapshots obtained for yeast heat shock stress regulatory modules 39 and 151 over time (0’, 5’, 15’, 30’ and 60’), highlighting the ranks of regulators exhibiting large relevance variations. In every row, each figure highlights the relevance of a particular regulator placed among the 30 best ranked TFs for a specific time point. Regulators appear in the top semi-circle from left to right in decreasing order of ranking score, and target genes are shown in the bottom semi-circle. Orange and green arcs respectively identify “regulates" and “regulated by" relations for the highlighted regulator. From top to bottom, first and second rows expose the ranks of Mig1p and Rim101p targeting the genes in biclusters 39, while third and fourth rows expose the ranks of Hcm1p and Arr1p in bicluster 151.

We finally analyzed the variation of the transcription factor relevance over time output by Regulatory Snapshots. We focused on four of the most varying TFs in terms of relevance scores, from those included within the top 30 and appearing among the 20 best ranked in at least one of the time points: Mig1p and Rim101p for bicluster 39, and Hcm1p and Arr1p for bicluster 151 (Figure 6). Mig1p had its importance increased from 0’ to 5’ and from 30’ to 60’, coinciding with the abrupt up and downregulation of the target genes in bicluster 39, respectively (Figures 4 and 6). This agreement is consistent with the documented role of Mig1p as a transcriptional repressor and its involvement in the negative regulation of gene-specific transcription from RNA polymerase II promoter [39]. The increase in the relevance of Mig1p relative to its absolute expression value was most significant at 0’, suggesting maximum activity of TF role at this time point (see Supp. Material). This is plausible both given its repression ability and considering that most of its target genes were downregulated at the time (Figure 4). Contrary to Mig1p, Rim101p had its relevance significantly decreased between 0’ and 5’ and 30’ and 60’. From 5’ to 30’, however, this TF known to be involved in cell wall construction [39] gained several positions in the relevance scale, achieving rank 3. Hcm1p, the forkhead transcription factor driving S-phase specific expression of genes involved in chromosome segregation [39] in bicluster 151, had its relevance increased between 0’ and 5’ and between 30’ and 60’, in agreement with the sudden down and upregulation behavior of the target genes (Figure 4). Comparative analysis between the ranking scores and absolute expression values for Hcm1p further revealed consistent activity as a TF in all time points except at 30’, where it rather acted as a target (see Supp. Material). Arr1p showed a slight relevance decrease between 0’ and 5’, achieving its best rank 13 at 15’, which then maintained with residual variation until 60’.

Contrary to many available approaches, Regulatory Snapshots does not make assumptions of consistency between the expression of regulators and targets. Regulators may thus be selected even if their expression was not measured, as in the case of Aft1p. Notably as well, most regulators at the top of the ranking were not included in the biclusters and effectively yielded distinct patterns from their targets, namely Crz1p, Msn2p, Rpn4p, Sfp1p, Yap1p for bicluster 39, and Abf1p, Leu3p, Met4p, Ste12p, Yox1p for bicluster 151.

### Human Epithelial-to-mesenchymal Transition

We built a human regulatory network as in [25], using JASPAR position-specific scoring matrices (PSSMs) and UCSC Human Genome (hg19) sequences [45]. Matrix identifiers (UniProt) were mapped to their encoding genes (NCBI Entrez) and RefSeq sequence accession numbers (NCBI GRCh37, Feb 2009) were converted to Entrez. Matrices and sequences with unmapped identifiers were filtered. We used the sequences 200bp upstream and 0bp downstream the transcription start site. We matched the PSSMs against the sequences using the PoSSuM software [46] and filtered results below a *p*-value cutoff of 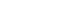. Edge weights were obtained by rescaling the raw matching score interval of each PSSM to 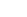 and selecting the highest scoring match for every PSSM-sequence pair. This generated a network with 50386 unique regulations and 18088 genes, from which 65 acted as regulators. In this study, we analyzed expression time series data obtained for human cells undergoing TGF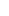-induced epithelial-to-mesenchymal transition (EMT) [27]. EMT is a fundamental process originally reported in embryonic development, which causes epithelial cells to: i) lose the adhesion structures that typically maintain them tightly together and largely immobile; ii) undergo cytoskeleton reorganization; and iii) acquire stemness and mesenchymal-like properties [47]. EMT has often been reported to resemble biological events responsible for promoting the migration and invasiveness of epithelial tumors (around 90% of all cancers [48]) to distant sites and thus leading to the development of metastasis. In the original experiment, expression levels were measured at 9 points over a 72 hour period (0h, 0.5h, 1h, 2h, 4h, 8h, 16h, 24h, and 72h) and preprocessed as described by Keshamouni et al. [27]. We further merged the replicates for each time point, converted the HGNC gene symbols for each Affymetrix probeset in the data to official HGNC and from these to Entrez Gene based on mapping files retrieved from the HGNC FTP repository, filtered genes with no valid or ambiguous conversion between both nomenclature sets, merged the expression values for probes denoting the same gene, and filtered genes absent from the human regulatory network.

#### Human EMT transcriptional modules

**Figure 7:**
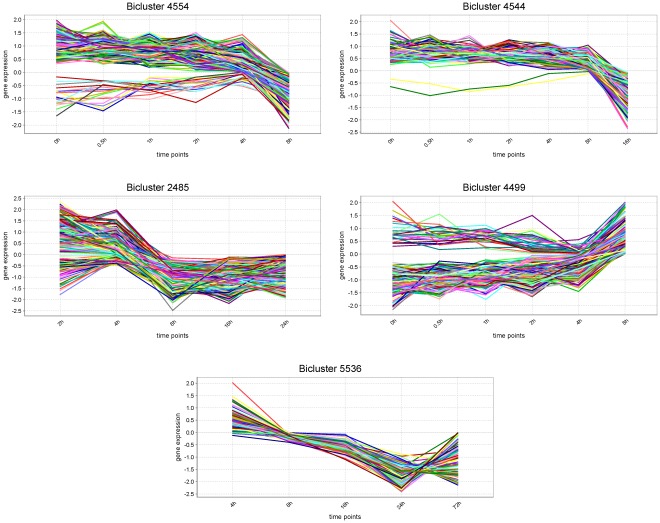
Expression profiles of five enriched human epithelial-to-mesenchymal regulatory modules. This figure shows the expression profiles of the genes in five of the regulatory modules obtained for the human epithelial-to-mesenchymal expression data and the human regulatory network containing regulations from the JASPAR database (modules 4554, 4544, 2485, 4499, and 5536), yielding some of the highest numbers of significantly annotated Gene Ontology terms. Expression levels were normalized by gene to zero mean and unit standard deviation.

We obtained biclusters and calculated the overrepresentation of Gene Ontology annotations following the same procedure used for the yeast dataset. Post-processing involved filtering biclusters containing less than 50 genes or less than 5 time points, and sorting in descending order of number of highly significant Gene Ontology terms (Bonferroni corrected *p*-value 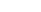). Among the first 50 biclusters we observed the emergence of five major groups of biclusters, yielding different patterns but showing consistent abrupt expression changes and similar functional properties. Notably, none of these biclusters encompassed all the time points in the experiment. This contrasts with the case study in yeast, in which the temporal window of the experiment was well delimited for the problem, therefore generating a reasonable number of clusters (spanning all conditions). We focus our analysis on a representative bicluster for each of the five major EMT groups (Figure 7). Bicluster 4554 was associated with oxidation-reduction regulation within the cell cycle. The genes in this bicluster revealed a relatively stable expression level during the first 4 hours, followed by an abrupt decrease between 4h and 8h after injection with TGF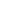. This is consistent with EMT alterations in the redox control of the cell cycle leading to increased invasiveness in tumor progression stages [47]. Genes in bicluster 2485 were linked to telomere organization, ncRNA metabolism and DNA replication. They exhibited a strong reduction in their expression levels also between 4h and 8h, denoting a potential inhibition of telomerase activity consistent with experimental evidence [49]. Telomerase activity is key to the immortalization (and proliferation) of tumors and cancer stem cells are known to exhibit telomerase expression [50]. Nevertheless, there is evidence that EMT bypasses cellular senescence to some extent via alternative mechanisms [49], while mesenchymal stem cells tend to show very low or undetectable telomerase levels [50]. Bicluster 4544 comprised genes implicated in cellular amino acid, lipid, and aldehyde metabolism. The drastic decrease in the expression level experimented by these genes between 8h and 16h is in accordance with the growth inhibition and reprogramming of metabolism, opposed to an increase in mobility and invasiveness potential, experimented by the cells undergoing EMT [51]. The effects of growth arrest were further observed in bicluster 5536, significantly annotated with cell division and chromosome segregation, whose genes showed a steady decrease in expression along a 20 hour period, between 4h and 24h. Genes in bicluster 4499 expressed coherently during the first 8h of the experiment and were associated with cellular component movement, locomotion, localization and cell junction organization, as well as cytoskeletal protein and calcium ion binding. Both these functional properties and the abrupt increase in expression exhibited by the genes between 4h and 8h after EMT induction effectively confirm the transition undergone by the cells from epithelial-to-mesenchymal phenotype.

#### Human EMT regulatory modules

Similarly to the case study in yeast, we obtained rankings of regulators for each of the five EMT-related transcriptional modules (biclusters) at every time point. In this case, the input for TFRank consisted in the expression levels measured for genes undergoing TFG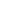-induced EMT at the time points in each bicluster and the human network of regulatory associations. Unfortunately, the human regulatory network is far from complete, containing a limited number of transcription factors. This resulted in very similar rankings that often included regulators known to target a large number of genes and therefore participate in a broad range of biological processes. In this context, we decided to focus on the transcription factors appearing at least once in the top 10, considering the rankings obtained for all the time points in each bicluster. We then identified some transcription factors for each bicluster among those that had showed up less among the top 10 in all the five biclusters, under the assumption that these would be more specific for the biclusters where they ranked high. For bicluster 4554, this procedure highlighted *PLAG1* and *MZF1*, which were attributed maximum relevance at 8h and 4h, respectively. Both regulators have been reported as inducers of tumor metastasis via the regulation of specific genes or pathways implicated in metastatic forms of cancer [52], [53]. Transcription factors *E2F1* and *IRF1* were selected for bicluster 2485. Interestingly, the expression of both *E2F1* and *IRF1* dropped between 4h and 8h, following the tendency of the genes in the bicluster, although their relevance has increased in this time frame. The evidence of *E2F1* and *IRF1* downregulation in this experiment suggests the silencing of their roles as metastasis suppressors [54], [55]. For bicluster 4544, the above procedure selected *FOXA1*, *FEV* and *HNF1B*. These transcription factors registered local maxima of importance before 2h for *FOXA1* and *FEV*, and at 2h for *HNF1B*, and later at 8h (*FOXA1*) or 16h (*FEV* and *HNF1B*). While the relevance on the first interval seems to be influenced by the upregulation of the genes encoding these transcription factors, the latter is more likely due to the drastic change exhibited by the target genes in the bicluster, as variations in relevance and expression were not consistent at those time points. Notably, *FOXA1* is a known negative regulator of epithelial-to-mesenchymal transition and all three transcription factors are involved in cell differentiation, organ morphogenesis and development characteristic of EMT. Five transcription factors, *IRF2*, *SRY*, *CREB1*, *NKX3-1* and *NFIL3*, appeared in the top 10 exclusively in rankings obtained for bicluster 5536. Genes *IRF2* and *SRY*, related to cell proliferation and cell differentiation, respectively, were considered most relevant at the first and last time points of the bicluster time frame (4h/8h and 72h), eventually relating to before and after the cellular reprogramming during EMT. The remaining regulators, *CREB1*, *NKX3-1* and *NFIL3*, exhibited a steady increase in relevance between 4h and 16h. This variation inversely proportional to the changes observed in the expression level of the genes in the transcriptional module, which could explain an eventual repressor control exerted by the three factors upon these targets. Functionally, the roles of *CREB1*, *NKX3-1* and *NFIL3* in the regulation of cell cycle, circadian rythm and organism growth, are consistent with the annotations yielded by the target genes and with the expression evidence of growth arrest experimented by the cells during EMT. In bicluster 4499, *NHLH1* arose as a relevant player. This transcription factor possesses documented interactions with major regulators of EMT, such as *TFC3*, and with several genes encoding cysteine-rich proteins containing LIM domains, of which *CSRP3* is probably the most relevant [56]. Participating in cell growth and somatic differentiation, *CSRP3* is also involved in the regulation of cellular calcium ion concentrations affecting the cadherins, important mediators of cell-cell adhesion and cytoskeleton organization [57].

### Comparison with State of the Art Tools

Available tools for the identification of regulatory modules can differ significantly in input data, definition of module, relationships within and between modules, and output. Systematical comparisons are thus either unfeasible, or likely to be performed in such terms that will favor a particular method in detriment of the others. In this section, we compare Regulatory Snapshots with a recent contribution to regulatory network inference, namely Physical Module Networks (PMN) [9]. PMN applies a learning procedure similar to that of Module Networks (MN) [8], alternating between two optimization steps at each iteration: i) a rearrangement of the network structure explaining the expression profiles of the genes in each module relative to the current module partitioning, and ii) an update of the module assignment relative to the current regulatory network structure. PMN and MN describe the data based on Bayesian models and derive an evaluation score from its posterior probability. Greedy hill-climbing search is then used to identify high scoring assignments or structures.

Simultaneous optimization of transcriptional control and response, performed by PMN and MN, seems theoretically preferable to the strategy of Regulatory Snapshots, which first groups genes in modules based exclusively on expression and then identifies regulators through integrated analysis. Nevertheless, Regulatory Snapshots showed very good performance with minimal guidance. Its strength lies in its prior search for temporal expression patterns, which delivers more specific and functionally coherent modules per se than other available clustering approaches [17]. First, it effectively finds the best solution, namely all maximal temporal transcriptional modules. Second, it focuses on local coherent responses, known to prevail in most interesting cases of transcriptional response [1], [18]. Third, it incorporates time dependency. Fourth, it allows genes to belong to more than one module (modules can overlap), and thus participate in distinct biological tasks with different partners over time. This setting presents more realistic assumptions towards transcriptional response than general purpose clustering techniques, such as those employed by PMN and MN. Clustering fits global models to expression data (consistency across all time points), often looking for disjoint groups, and require a predefined number of clusters. These restrictions tend to generate artificial partitions of the data that deviate from their natural organization, and ultimately lead to clusters lacking enriched functional annotations [1], [9]. Similarly, as the authors further observe, the optimization procedure is highly dependent on the initial cluster assignment and susceptible to converge to local maxima [8].

Likewise, the PMN formulation restricts the configuration of the regulatory pathways underlying a particular transcriptional response. Typically a single path is selected per module, consisting of an indirect regulator linked by a physical interaction pathway to a direct regulator exerting transcriptional control upon the consistently expressed genes. One drawback of this scheme is that it ignores that gene response is more likely the result of a combined effect of multiple regulatory players and pathways than the isolated action of a given transcription factor [25]. Also, the role of direct regulators is prone to be overtaken by transcription factor hubs, given the criterium to maximize the number of direct targets within the module. As in Module Networks [8], it is assumed that the regulator exhibits an expression profile similar to the one of its targets, a constraint that does not hold in most datasets. Contrary to separately assessing indirect and direct roles of regulators, Regulatory Snapshots calculates a measure of relevance which naturally embeds direct and indirect control exerted upon the targets and incorporates full network topology (all paths) on a more systemic and integrated view of gene regulation. It further provides mechanisms to mitigate the hub effect, through normalization, in the context of both regulation weights and final ranking score [25].

In essence PMN has been shown to perform well using data previously isolated relative to a particular biological process [9]. However, any pathway selected from the network in such context is likely to be pertinent to the problem to some extent. This leads us to the observation that PMN and Regulatory Snapshots serve distinct purposes. PMN focuses on reconstructing pathways between regulators and targets known to be involved in the biological events under study, depending heavily on established knowledge. Specifically, it requires input lists of putative indirect and direct regulators, in addition to protein-protein interactions, protein-DNA interactions, and expression data. In contrast, Regulatory Snapshots infers the biological context exclusively from the expression data and traverses the affected part of the context-free regulatory network to automatically rank relevant transcription factors. It is therefore applicable to cases where prior information is scarce and tailored to unravel novel hypotheses from high throughput data. Concerning the type of interactions, PMN considers both protein-protein interactions and regulatory (protein-DNA) interactions. Including evidence of physical interactions is likely to mitigate issues caused by limited availability of regulatory information, on which Regulatory Snapshots exclusively relies. On the other hand, the method can no longer guarantee that the pathway built between indirect and direct regulators possesses in fact a regulatory nature.

Not surprisingly, both methods lack full characterization of the dynamic nature of gene regulation. It is known that only a subset of the regulatory interactions in the network underlying a particular transcriptional response are in fact involved in the biological process under study and that the group of active interactions changes over time, as more specific tasks occurring in the cell start and finish. Not only this increases the complexity of the problem, as also little or no large scale experimental information exists on dynamics of interactions. PMN regards the network as static and identifies the part which best describes the behavior of the genes at all time points. In this regard, PMN analysis outputs a single network topology, in which the temporal dimension is lost. Regulatory Snapshots performs an analysis per time point, generating a list of transcription factors ranked according to a measure of relevance of those regulators relative to the response observed at such time point. In this context, we put forward a novel way to interpret dynamics and highlight the variation of transcriptional control over time.

On another note, Regulatory Snapshots strategy is fast and highly scalable, accommodating well for large expression datasets and interaction networks. The worst case time complexity for a complete analysis of the data is 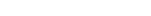, where 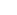 and 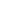 denote the numbers of genes and time points in the expression data, 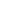 is the number of biclusters (transcriptional modules) to be further inspected for an underlying regulatory network, 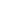 is the total number of interactions in the context-free regulatory network graph, and *N* is the number of iterations for the transcription factor ranking procedure (typically, a value in the order of 10 will be sufficient [25]). The number of biclusters 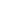 can be 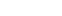 in the worst case. In practice, for real datasets it tends to be considerably smaller. Additionally, given its modular nature, Regulatory Snapshots allows the researcher freedom to filter uninteresting sets prior to the application of the second step. Several methods to filter and sort the biclusters according to different criteria have been previously made available and proved effective [17], [37]. Approaches like PMN and MN, or the related MEDUSA, are computationally intensive. In a recent study, MEDUSA was reported to take longer than 4 weeks to analyze a dataset containing 7000 genes using 1000 iterations. A parallelized version, fastMEDUSA, would be able to process the data about 40 times faster, using 100 processors, which would still acount for more than 2/3 of a day [58]. Significant reductions of the search space can be achieved through preselection of relevant data based on prior knowledge. However, this will make these methods unsuitable for automated and unbiased regulatory module discovery using high throughput data.

### Conclusion

We proposed Regulatory Snapshots, an integrative method to unravel and characterize regulatory modules of genes exhibiting coherent expression trends and their most relevant regulators over time. It defines a robust integration strategy for the problem while addressing the major concerns associated with current regulatory module identification methods. In particular, it effectively considers a temporal dimension that has been insufficiently exploited. Regulatory Snapshots is further able to combine prior knowledge with experimental data, integrate evidence of both regulation and function, and embed mechanics and dynamics, while incorporating time dependency, and consider systemic and individual features.

In a first step, biclustering is applied to identify coherent transcriptional responses in expression time series (CCC-Biclustering) [17]. We use an exhaustive approach which therefore guarantees to find all maximal subsets of genes exhibiting consistent expression profiles along subsets of consecutive time points. Although in some cases a large number of biclusters may be discovered, several numerical and statistical criteria can be used in order to filter and sort the resulting gene modules [37]. On the other hand, a biclustering strategy enables, but does not restrict itself to, the search for local expression patterns known to prevail in transcriptional responses. Global patterns are also discovered when they exist. Similarly to most algorithms for analysis of expression data, CCC-Biclustering relies on a given number of classes to express different activation levels, causing results to be influenced by the use of a discretization method. Nonetheless, it has been shown that discretization techniques based on transitions between time points are appropriate for the analysis of expression levels and can present an advantage to using real-valued data, as they reduce the complexity of these large data and enable to discard non-significant differences between the expression levels of different genes or time points due to natural conditions or technical measurement details [17], [35]. We further support the choice of three states based on the following observations: researchers are often interested in describing expression trends using only two or three distinct activation levels (we used three); the choice of the discretization threshold is made dependent on the parameters of the preceding normalization step to ensure profile comparability.

In a subsequent step, personalized ranking is applied to determine the most relevant regulators exerting control upon the genes in a module at each time point (TFRank) [25], whereby an initial preference signal comprising the expression levels of the targets is diffused through the transpose of the regulatory network graph to devise a score for every TF. Dynamic and static properties of regulatory mechanisms are captured by straightforwardly incorporating transcriptional response and interactions into the score calculation. The ranking strategy is further able to perceive relevant regulations within a given biological context based on a combination of full regulatory connectivity and individual behavior. Relevance scores embedding indirect associations such as this one have been shown to be more informative and robust, outperforming measures based on direct interactions in a recent study on network-based disease candidate gene prioritization [59]. Overall, the personalized ranking framework presents a flexible solution that provides a number of features allowing for a fine tuning of the scores, including adjustment of regulations’ weights and initial signal, as well as control over preference for closer or farther regulations.

We used Regulatory Snapshots to study *Saccharomyces cerevisiae*’s response to heat shock and human epithelial-to-mesenchymal transition. In both case studies, the targets in the regulatory modules were found to yield coherent transcriptional profiles and functional properties. Results further confirmed the successful identification of TFs known to participate in the regulation of the modules. Additional TFs unraveled by Regulatory Snapshots underlied annotations consistent either with the biological process under study or with functional annotations enriched for the set of target genes. Some snapshots revealed coincident variations in the relevance of prominent TFs and the expression of their target genes in regulatory modules. In addition, we observed that the relevant TFs could be identified even though they did not exhibit expression coherence with their targets. Regulatory Snapshots thus proved effective to enable temporal exploration of regulatory networks and suitable for enhancing their dynamic properties. In particular, the underlying ranking scores suggested inherent ability to discern the primary role of a given gene at each time point, whether TF or target. Ultimately, the fact that results output by a largely automated approach with minimal guidance could be confirmed by prior knowledge supports the value of this integrative contribution to the study of regulatory networks over time through the identification of regulatory modules using expression time series and regulatory associations.

Several directions arise for future research. It is known that consistent expression profiles are not sufficient guarantee of co-regulation [60]. In fact, different genes regulated by non-overlapping sets of TFs may exhibit similar expression profiles and consequently be grouped into the same regulatory module. This discrepancy is sometimes revealed through functional enrichment with GO terms which are apparently not related. The opposite problem can also be observed, by which different biclusters that potentially overlap to some extent pertain very similar annotations and should eventually be grouped into larger structures (meta-biclusters). An improvement to the current Regulatory Snapshots strategy could involve the integration of additional information extracted from Gene Ontology graphs. Other highly desirable features of a temporal module discovery algorithm would include ability to infer and incorporate the status or level of intensity and direction, as well as the type of action upon a target, of each regulatory interaction, which presents a very complex task and can therefore be considered as a long term goal [61].
